# Red Wine, Resveratrol and Atrial Fibrillation

**DOI:** 10.3390/nu9111190

**Published:** 2017-10-30

**Authors:** Laura Siga Stephan, Eduardo Dytz Almeida, Melissa Medeiros Markoski, Juliano Garavaglia, Aline Marcadenti

**Affiliations:** 1 Postgraduate Program in Health Sciences: Cardiology, Institute of Cardiology/University Foundation of Cardiology (IC/FUC), Princesa Isabel Avenue, 370, Porto Alegre RS 90620-001, Brazil; emaildalaura@gmail.com (L.S.S.); edudytz@terra.com.br (E.D.A.); melissa.markoski@gmail.com (M.M.M.); 2 Postgraduate Program in Nutrition Sciences, Federal University of Health Sciences of Porto Alegre (UFCSPA), Sarmento Leite Avenue, 245, Porto Alegre RS 90050-170, Brazil; julianogaravaglia@gmail.com; 3 Institute of Technology in Food for Health, University of Vale do Rio dos Sinos (UNISINOS), Unisinos Avenue, 950, São Leopoldo RS 93022-750, Brazil

**Keywords:** atrial fibrillation, wine, trans-resveratrol

## Abstract

Atrial fibrillation (AF) is a common cardiac arrhythmia that is associated with increased risk for cardiovascular disease and overall mortality. Excessive alcohol intake is a well-known risk factor for AF, but this correlation is less clear with light and moderate drinking. Besides, low doses of red wine may acutely prolong repolarization and slow cardiac conduction. Resveratrol, a bioactive polyphenol found in grapes and red wine, has been linked to antiarrhythmic properties and may act as an inhibitor of both intracellular calcium release and pathological signaling cascades in AF, eliminating calcium overload and preserving the cardiomyocyte contractile function. However, there are still no clinical trials at all that prove that resveratrol supplementation leads to improved outcomes. Besides, no observational study supports a beneficial effect of light or moderate alcohol intake and a lower risk of AF. The purpose of this review is to briefly describe possible beneficial effects of red wine and resveratrol in AF, and also present studies conducted in humans regarding chronic red wine consumption, resveratrol, and AF.

## 1. Introduction

Atrial fibrillation (AF) is the most common cardiac arrhythmia and has been associated with increased risk for ischemic heart disease, major cardiovascular events, stroke, heart failure, chronic kidney disease, peripheral arterial disease, sudden cardiac death, cardiovascular, and all cause mortality [1]. Worldwide, in 2010, the age-adjusted prevalence of AF reached about 33.5 million individuals (0.5%) [2]; thus, the awareness of AF risk factors and the improvement of therapeutic approaches may contribute for its complex management.

Excessive alcohol intake, whether binge drinking or long-term abuse, has been associated with AF and other cardiac arrhythmias, but this correlation is less clear with light and moderate drinking [3,4,5,6,7,8,9]. The pathophysiology behind AF onset after binge drinking (“holiday heart”) is not entirely elucidated and is likely multifactorial, encompassing direct (cytotoxic) and indirect (increased sympathetic and parasympathetic activity) mechanisms. It has been postulated through animal models that both QT interval prolongation and shortening of the atrial effective refractory period might be related to AF onset after binge drinking [7,10]. Long-term abuse, on the other hand, is associated with left atrial enlargement and remodeling, which creates an anatomic substrate for AF [11].

Red wine consumption has been studied since 1981, when the French paradox of less cardiovascular diseases in spite of higher alcohol consumption was discovered. Beyond its well-known cardioprotective properties [12], low doses of red wine may acutely prolong repolarization and slow cardiac conduction in healthy subjects [13]. Resveratrol, a bioactive polyphenol that is found in grapes and red wine, has been linked to beneficial effects on cardiovascular diseases [14] and may exhibit antiarrhythmic properties [15,16]. However, it is not known if the antiarrhythmic effects of resveratrol can counterpoise the proarrhythmic effects of alcohol intake.

Particularly in AF, there are still no conclusive clinical trials proving that resveratrol supplementation leads to improved outcomes. Thus, the purpose of this review was to briefly describe possible beneficial effects of red wine and resveratrol in AF, and also present studies conducted in humans regarding resveratrol/chronic red wine consumption and incident AF.

## 2. Red Wine and Resveratrol

Wine is a complex mixture of several hundred compounds, many of them found at very low concentrations [17]. In general, the average concentrations of the major components of wine are: water, 86%; ethanol, 12%; glycerol and polysaccharides or other trace elements, 1%; different types of acids, 0.5%; and, volatile compounds, 0.5% [18]. The alcohol content varies widely among wines (from 10% to 14%) and is mainly obtained by yeast-converting sugars [19]. Glycerol, found at concentrations reaching the range of 10 g/L in red wines, is produced during alcohol fermentation and is influenced by the yeast strain, temperatures, pH, and oenological practices [20]. Tartaric acid is the most prevalent organic acid in wine, followed by malic and citric acids [19].

Red wine is different from other alcoholic beverages due to its content in various phenolic compounds [21]. During the winemaking process, polyphenols are the main phenolic compounds extracted from grapes, initially obtained by crushing the fruit, and intensified by the maceration and pumping over processes during fermentation [12]. Red wine contains both grape polyphenols (including proanthocyanidin, anthocyanin, phenolic acids and resveratrol) and phenolic compounds formed during alcoholic fermentation and wine ageing [22]. The amount of phenolic compounds in red wine may differ considerably according to the geographical area of production, the kind of vine, and the enological methods adopted for its production [23,24]. The antioxidant activity of red wine is strongly correlated with its total phenol content [25].

Resveratrol (3,5,4′-trihydroxy-trans-stilbene) is the most common phenolic compound present in grapes and red wine (average 1.9 ± 1.7 mg of trans-resveratrol/L, ranging from non-detectable levels up to 14.3 mg/L) [26]. The amount of resveratrol in wine is related to the permanence of the grape skins at the fermentation process; so, its concentration is significantly higher in red wine than in white wine [27]. Resveratrol is a stilbene derivative detected in grape skin and seeds [28]; although it has been considered the major functional compound in red wine [18], the amount of resveratrol is lower than other polyphenols (0.80 to 4.61 mg/L) when compared to caffeic and syringic acids, for example [25].

Beneficial effects of resveratrol on human health are well known. Trans-resveratrol has been one of the most extensively studied non-flavonoids due to its cardioprotective properties [14], anti-inflammatory, antibacterial, antifungal, antiviral, neuroprotective, antiproliferative, and anti-angiogenic activities [29]. However, the possible effects of resveratrol on cardiac rhythm and function are poorly understood.

## 3. Red Wine, Resveratrol and Biological Properties Related to Atrial Fibrilation

The actions of the red wine and its constituents on the cardiovascular system, for good or for bad, are well known [12,30]. Nevertheless, to discuss about the biological action of wine on AF, it is necessary to evaluate its components separately, focusing on the balance between pro and anti-arrhythmic activity and specific molecular mechanisms. In addition, it is important to consider that despite the complex and aging-related clinical risk factors for the disease, AF is heritable. Family history is well related, for example, to potassium or sodium ion channel mutations, to alterations in atrial natriuretic peptide genes, genes encoding transcription factors, those from GATA family (involved in cardiogenesis), homeobox genes (involved in cardiac development), and ryanodine receptors (responsible for releasing intracellular stores of calcium) [31]. Thus, although studies on biological functions of the phenolic compounds or alcohol present in the red wine on AF are somewhat scarcer, clarification on the relationship between these components and mutations that cause AF, allow us to postulate some observations.

Delayed afterdepolarizations constitute the most important mechanism of focal atrial arrhythmias. They result from abnormal diastolic leak of calcium from the sarcoplasmic reticulum, the main cardiomyocyte calcium storage organelle, through ryanodine receptors [32]. These repetitive delayed afterdepolarizations, which are in turn, generated by an anomalous genetic control cause focal atrial tachycardias. In this way, it has already been shown that resveratrol can act as an inhibitor of intracellular calcium release, via ryanodine receptors, through the activation of AMP-activated protein kinase (AMPK) enzyme [33]. This action would assist in the regulation of mitochondrial biogenesis and in the balance of the cardiomyocyte function. A study conducted in rats also showed that resveratrol was able to fix the calcium handling proteins in the sarcoplasmic reticulum to preserve contractile function in those animals subjected to pressure overload and contractile dysfunction [34]. In addition, Qian et al., 2012 also demonstrated in rabbits that resveratrol has an inhibitory effect on the late sodium current, a factor involved with abnormal repolarization and consequent arrhythmia [35]. Authors concluded that this effect reduces the sodium and calcium intracellular concentration, as well as potentially eliminating the calcium overload, and ultimately inhibiting the electrical abnormalities. Still, on this subject, an important atrial-specific target is the voltage-gate potassium channel Kv1.5, expressed in the atria, but not the ventricles, and coupled to the late sodium current overactive channel, inflammation/oxidative stress, and activation of the nuclear factor of activated T cells (NFAT) all have been implicated in the development of AF [16]. Finally, in this matter, a study conducted in rabbits showed that resveratrol could revert an abnormal signaling, via gene expression activation of phosphoinositide 3-kinase (PI3K)/Protein kinase B (Akt)/endothelial nitric oxide synthase (eNOS) axis, which play a role in the inhibition of pathological signaling cascades in AF, and hence correcting the action potential duration, causing a suppression on AF [15].

However, despite the benefits that resveratrol and other phenolic compounds and even the alcohol present in red wine can bring to the cardiovascular system and the control of atrial rhythm function, the excessive consumption has a strong relation with AF [4,5]. In this context, Maki et al., 1998 connected ethanol intoxication in patients with ethanol-induced atrial fibrillation with an increased beta-adrenoceptor density, suggesting an increased sympathetic stimulation [36]. In contrast, Churchill et al., 2008 claimed that the damaging effect of alcohol present in the wine on the cardiac cell function is related to the duration of exposure to ethanol [37]. This effect would be related to an ability of ethanol action on membrane receptors and signaling triggered that causes the activation of ATP/potassium pumps (target of several genetic mutations) in the mitochondria. Therefore, as a “protective” effect, the activation of these mitochondrial potassium channels would increase the volume and the mitochondrial metabolism, reducing the calcium overload and oxidative stress. But in excess or in chronic exposure, cellular receptors would be desensitized and mitochondrial biogenesis could not occur, leading to an imbalance of intracellular calcium. Further, by promoting oxidative damage to multiple subcellular and cellular structures, reactive oxygen species have been shown to induce the intra and extracellular changes necessary to promote the pathogenesis of AF [38]. In this way, resveratrol present in red wine is notable in promoting activation of anti-oxidative mechanisms [12].

Finally, something that would be very useful to the understanding of the components of red wine on the cellular function and biological processes in AF would be the experiments of genome-wide profiling and systems biology [39], because these technologies assist in the understanding of complex diseases as a whole. These findings also could benefit clinical studies, which are scarce for use of red wine, resveratrol and AF.

### Studies in Humans

An electronic search in PubMed was performed using the combination of terms “wine and atrial fibrillation” and “resveratrol and atrial fibrillation”. After reviewing the first results, we included an additional search of the combination of terms “alcohol and atrial fibrillation”, only in the titles. The results were then filtered by species (humans) and language (English). Thirty-seven distinct articles from this search were obtained. Most of the studies that evaluated alcohol consumption as a risk factor for AF did not specify the alcoholic beverage subtypes. Papers focusing on acute alcohol intake and AF were not included. Data about categories of wine consumption and the adjusted hazard ratio (HR) or relative risk (RR), as well as the adjustment variables were collected. For discussion purposes, we used National Institute on Alcohol Abuse and Alcoholism definition for heavy drinking: >14 drinks/week for men and >7 drinks/week for women and adults over 65 years of age [40].

Four articles relevant for this review (related to new-onset AF) were identified (i.e., that mentioned specific wine consumption or remarked that the preferred source of alcohol did not change the results). Table 1 summarizes the main aspects of these studies, as well as the adjusted HR or RR for wine consumption and risk for AF. We did not find randomized clinical trials regarding resveratrol and AF, nor cohorts that evaluated specifically the consumption of red wine and AF.

Frost et al. [41], in 2004, examined 47,949 participants (mean age, 56 years) in the Danish Diet, Cancer, and Health Study. During a mean follow-up of 5.7 years, AF or flutter developed in 374 men and 182 women. After adjusting for established risk factors, there was a modest increase in risk of AF by increasing the alcohol consumption in men; in women, despite HR suggesting increased risk, there was lack of statistical significance. When using the lowest quintile of alcohol consumption, adjusted hazard rate ratios in men in quintiles 2, 3, 4, and 5 were 1.04, 1.44, 1.25, and 1.46, respectively (*p* for trend, 0.04). The authors did not disclose data about the preferred source of alcohol (wine, beer, or spirits), but they mentioned that this variable did not change the aforementioned associations.

Mukamal et al. [4], in 2005, investigated a cohort of 16,415 participants enrolled in the Copenhagen City Heart Study. Patients were excluded from analyses if they had cardiovascular disease (defined by self-report of hypertension, stroke, coronary artery disease, or use of cardiovascular medications). They ascertained use of beer, wine, and spirits individually. A total of 1071 cases of AF occurred during follow-up (mean 17.4 years). The consumption of 35 or more drinks per week among men was associated with a HR of 1.45 (95% confidence interval (CI) 1.02 to 2.04). There was no statistically significant association found for women. In a subgroup analysis for individual alcoholic beverages, none of the three beverages was associated with increased risk of AF.

Mukamal et al. also studied this association in the United States in 2007 [42], as part of the Cardiovascular Health Study, a population-based cohort of adults 65 years and older. A total of 5609 participants reported their use of beer, wine, and spirits yearly and 1232 cases of AF were documented during a mean follow-up of 9.1 years. Intake of any specific alcoholic beverage was not associated with development of AF.

Larsson et al. [43], in 2014, studied a cohort of 79,019 Swedish men and women. It was shown that the consumption of more than 14 drinks/week of wine was associated with an increased risk of AF (HR for wine 1.08–1.68, *p* for trend 0.01). Consumption of liquor, but not beer, was also associated with an increased risk. The same pattern of association was found among binge drinkers: liquor and wine, but not beer, were associated with increased risk of AF in a multivariate analysis.

## 4. Discussion

Studies in humans suggest that alcohol consumption is associated with an increased risk of AF among heavy drinkers only. Wine consumption, when compared to other beverages, did not alter the risk for new onset AF across different strata of alcohol intake. This was true both for chronic alcohol intake and for binge drinking. In the largest of the studies, both wine and liquor groups had more incident AF cases when compared to beer, but no potential explanation for this finding was given.

These findings indirectly questions the antiarrhythmic effects of resveratrol in humans or, at the very least, its ability to counter the proarrhythmic effects of alcohol itself. It should be noted, however, that none of these studies assessed the type of wine consumed. Since red wine contains a much larger amount of resveratrol, determining the type of wine consumed would have provided better indirect evidence on resveratrol association with AF. Furthermore, participants were categorized into the each group according to the beverage that they consumed the most, which means that other beverages were likely consumed as well. This could negate any beneficial effects of wine intake, especially smaller ones.

Resveratrol is present mainly in grapes, red wine, and in very low concentrations in peanuts, pistachios, berries, tomatoes, chocolate, apples, and beer [44,45]. Its beneficial effects in humans are dose-dependent [46], but it is suggested that higher doses (single or multiple daily doses up to 600 mg/day) may be safe [47,48]. In addition, repeated oral administration of high daily doses of *trans*-resveratrol is well tolerated, reflecting in relatively low plasma concentrations of *trans*-resveratrol [46]. It is known that daily doses of resveratrol from 0.5 to 1.0 g may exert pharmacological effects [47], being a potential alternative to non-steroidal anti-inflammatory drugs and selective cyclooxygenase (COX) inhibitors, for example [46]. Thus, and considering that red wine has a small amount of resveratrol per liter, the amount of wine that would be required for resveratrol exert pharmacological effects would be very large; in this case, the harmful effects of excessive alcohol consumption would be pronounced.

Several limitations apply to the studies in this review. Self-report of wine consumption brings potential bias, since both over and underreport may occur. As far as memory bias goes, those individuals with incident AF might be more prone to remember and report alcohol consumption, since this is a well-known risk factor for this arrhythmia and is likely questioned by physicians whenever AF is diagnosed. On the other hand, since red wine has long been popularly associated with better cardiovascular health, it is possible that those individuals with a preference for red wine consumption have more health conscious habits.

## 5. Conclusions

Red wine and resveratrol may exert beneficial effect on AF, despite that its mechanisms are poorly understood. Clinical studies on the intake of red wine specifically and/or resveratrol could provide additional information regarding this issue, and even for complementation of therapeutic approaches.

## Figures and Tables

**Table 1:** Characteristics of Prospective Studies of Wine Consumption and Risk of Atrial Fibrillation.

First Author, Year, Country, Reference and Study Design	Sex, Age Range (Years)	Follow up (Years)	N of Participants/N of AF Cases	Categories of Wine Consumption	Adjusted HR/RR (95% CI)	Adjustments
Frost, L., 2004, Denmark, cohort * [41]	Men, 50–64	5.7	22,528/374	4.1 g/day	1.00 (reference)	Age, education, BMI, height, smoking, systolic BP, treatment for hypertension, and total serum cholesterol
				12.1 g/day	1.04 (0.73–1.49)	
				20.0 g/day	1.44 (1.04–2.01)	
				36.1 g/day	1.25 (0.89–1.76)	
				68.7 g/day	1.46 (1.05–2.04)	
Frost, L., 2004, Denmark, cohort * [41]	Women, 50–64	5.8	25,421/182	1.1 g/day	1.00 (reference)	Age, education, BMI, height, smoking, systolic BP, treatment for hypertension, and total serum cholesterol
				4.6 g/day	1.09 (0.68–1.75)	
				9.4 g/day	1.27 (0.80–2.04)	
				15.6 g/day	1.23 (0.77–1.98)	
				38.8 g/day	1.14 (0.70–1.85)	
Mukamal K., 2005, Denmark, cohort [4]	Men 26–75	16.6	7366/548	<1 drinks/week	1.00 (reference)	Age, education, BMI, height, physical activity, smoking, cohabitation, family history of CVD, DM, income, FEV1, use of BP medication, systolic BP, history of CHD and HF
				1–6 drinks/week	0.85 (0.69–1.05)	
				7–13 drinks/week	0.97 (0.67–1.39)	
				14–20 drinks/week	0.81 (0.45–1.43)	
				≥21 drinks/week	0.99 (0.46–2.13)	
Mukamal K., 2005, Denmark, cohort [4]	Women 26–73	18.3	7588/523	<1 drinks/week	1.00 (reference)	Age, education, BMI, height, physical activity, smoking, cohabitation, family history of CVD, DM, income, FEV1, use of BP medication, systolic BP, history of CHD and HF
				1–6 drinks/week	0.95 (0.76–1.19)	
				7–13 drinks/week	1.11 (0.75–1.65)	
				≥14 drinks/week	1.19 (0.55–2.57)	
Mukamal, K., 2007, United States, cohort [42]	Both ≥65	9.1	4502/1107	0 drinks/week	1.00 (reference)	Age, sex, race, income, height, waist circumference, physical activity, use of psychoactive medication, DM, hypertension, CHD, HF and total cholesterol level
				<1 drinks/week	1.13 (0.97–1.32)	
				1–6 drinks/week	1.04 (0.84–1.28)	
				≥7 drinks/week	1.06 (0.78–1.42)	
Larsson, S., 2014, Sweden, cohort [43]	Both, 45–83	12	68,848/6019	<1 drinks/week	1.00 (reference)	Age, sex, education, smoking, BMI, family history of MI, history of CHD or HF, DM, hypertension
				1–6 drinks/week	1.01 (0.96–1.07)	
				7–14 drinks/week	1.09 (0.97–1.23)	
				>14 drinks/week	1.30 (1.06–1.61)	

* Results are for consumption of alcohol, but the addition of information on the preferred type of alcohol (beer, wine, or mixed) did not changed the estimates. CVD = cardiovascular disease; BP = blood pressure; BMI = body mass index; DM = diabetes mellitus; CHD = coronary heart disease; HF = congestive heart failure; MI = myocardial infarction.
